# Involvement of Civil Society in India’s Polio Eradication Program: Lessons Learned

**DOI:** 10.4269/ajtmh.18-0931

**Published:** 2019-10

**Authors:** Roma Solomon

**Affiliations:** Secretariat Director, CORE Group Polio Project/India, Gurgaon, India

## Abstract

India achieved the title of a polio-free country in March 2014 after a prolonged battle with the poliovirus that threatened millions of children and paralyzed scores of them. Although there has been considerable documentation of the technical strategies applied over the years, not enough has been written on the other warfront that had opened, namely, the battle between the people and the polio eradication program. This article describes the immense people-driven challenges to the polio program and the need for tailor-made and novel responses. This is when the U.S. Agency for International Development–funded CORE Group Polio Project (CGPP)/India stepped in and started work in 1999. The project, a consortium of CORE Group member international non-governmental organizations (NGOs) and local NGOs, formed a bridge between communities and the government program. This article describes how CGPP/India listened to the families and communities who refused to participate in the polio eradication program and then strategically addressed their concerns. These lessons from India can benefit other public health priorities that require civil society involvement, as most public health efforts do.

## INTRODUCTION

In March 2014, India was declared polio-free, along with the entire Southeast Asian region, and the whole country cheered the historic and notable culmination of one of the longest public health initiatives to date.^1^ The captivating story behind India’s long fight against polio should be memorialized through the critically important lessons learned that are applicable to numerous initiatives for public health in the context of stubborn development and humanitarian issues.

As recently as 2009, India reported almost half the world’s polio cases—741 of a total 1,604 cases worldwide. It was commonly believed that India would be the last country to eradicate polio, if ever. However, a mere 2 years later, in January 2011, the last case was identified, and India was certified as polio-free in 2014.^2^

At the onset, the Polio Eradication Initiative kicked off with country-wide National Immunization Days in 1995. Women lined up at dawn with their babies at vaccination booths to get them vaccinated against polio. There was a festive air in every locality, hinting at the beginnings of a very successful “people-owned program.”

However, once the immunization data were examined, it became obvious that children were being missed in large numbers, jeopardizing the ability to meet immunization targets and ultimately the ability to achieve the herd immunity required to stop transmission. This realization led the government, in 1999, to take the bold step of sending vaccinators to each house to ensure that every child was reached.

Then the unexpected happened. Parents started shutting their doors on the vaccinators, refusing to allow their children to be vaccinated, and the enthusiasm of parents turned to reluctance in some states, and strong aggressive resistance in others. The government was faced with a new challenge: understanding why communities would turn against the very people who were trying to protect their children from a deadly disease that had crippled so many children in cities, villages, and slums.

Widespread resistance highlighted the frail and sensitive interface between the government and communities, particularly those in underserved areas.^3^ For families living in places where health systems were weak, this vertical program became viewed as coercive and as threatening as another similarly organized program from years before—family planning. Rumors circulated in communities, leading to suspicions about the vaccine. People started hiding their children, especially boys, fearing the vaccine would leave them impotent. Parents would lock up their houses and take their children elsewhere so that they could avoid being vaccinated. Auxiliary nurse midwives (ANMs) and other government frontline workers also were unwelcome. In one such instance, the author accompanied a CORE Group Polio Project (CGPP) team of mobilizers and an ANM to a house in Bhojpur block of Moradabad district, Uttar Pradesh, in 2004. The grandmother hid the male child in a chicken coop and threatened to kill him and the team with a large knife if the team stepped inside. Rumors and mistruths about harm caused by polio vaccine spread quickly from village to village or from one part of a town to another. *Fatwas*† started to appear claiming that the vaccine was *haram*,‡ causing more doors to be shut. As this resistance built, it became clear that if new strategies that involved communities were not devised, India would lose the war against polio.

## COMMUNICATION STRATEGIES

Since 1999, the CORE Group (a consortium of international and national nongovernmental organizations funded by the United States Agency for International Development, USAID) has been working on polio eradication, as described elsewhere in this series.^4^ Polio eradication has become one of India’s flagship programs in many states of the country. The CGPP, meanwhile, was using schoolchildren, nursing students, teachers, and community-based groups to mobilize mothers to bring their children for vaccination on booth days. Volunteers listened closely to the communities and when they faced resistance, sought ways to dispel mistruths and convince parents of the value of vaccines. At the height of resistance, the community mobilizers hired by the CGPP knocked at each door to gain entry into people’s houses, minds, and hearts to gain increased acceptance of the oral polio vaccine.

In the early days, Uttar Pradesh was the state with maximum opposition to the polio eradication program. The United Nations Children's Fund (UNICEF) and CGPP community mobilizers (described elsewhere in this series^5,6^) would approach households together for fear of being turned away, abused, or assaulted. In 2003, the CGPP and UNICEF jointly approached the Uttar Pradesh government with the proposal of forming a social mobilization network (SMNet). Mobilizers of both agencies would be given the same nomenclature and key responsibilities, and they would be supervised at block, district, and regional levels. Community mobilization coordinators (CMCs) were selected from the high-risk and most resistant communities identified by the government and the National Polio Surveillance Project. These CMCs, mostly women, were trained in interpersonal communication, negotiation skills, and other behavior change strategies and tasked with visiting households to explain to parents the importance of vaccination against polio and other diseases. The CMCs are paid volunteers, meaning that they receive a fixed honorarium, but this is not considered to be a formal salary. The honorarium was originally paid in cash, but more recently it has been paid via bank transfer. The use of the term “honorarium” reflects the temporary nature of this financial support.

In most cases, the process of convincing parents to accept polio vaccination was not as simple as a single visit to the house. To reach all target children, each house with children had to be marked and visited multiple times, even when faced with rudeness and resistance. During home visits, the community mobilizers spent time with women, explaining the advantages of immunization, dispelling mistruths, and discussing other health and sanitation issues. Repeated visits built more trusting and welcoming relationships with the families and within the communities.

When it was found that a particular section of society was resisting more than the others, a meeting was arranged with the *Shahi Imam* of Jama Masjid in Delhi.§ The *Shahi Imam* rarely gives such audiences, but it was possible to convey to him personally the growing resistance in families. He provided CGPP/India Director with contacts of some of his followers in Uttar Pradesh who could help gain access to imams and other priests. A few of these people still work for the polio eradication program, and their sustained support has proved increasingly valuable over the years. This strategy also paved the way for the entry of the so-called influencers or local leaders (licensed and unlicensed practitioners, priests of various religions, shopkeepers, teachers, and so forth) who would accompany the mobilizers trying to change resistor families into acceptor ones.

CMCs noticed persistent resistance in some households. Visits were marked by repeated excuses such as “My child is sick” or “She is too small/weak to be vaccinated.” Community influencers were invited to frequent meetings where CMCs gave information about the benefits of vaccines, dispelled myths, and taught negotiation skills to prepare them for conversations with resistant parents. The contribution of influencers was purely voluntary, but their sustained support and commitment produced a clear, strong impact on the communities they served.

**Figure 1.**
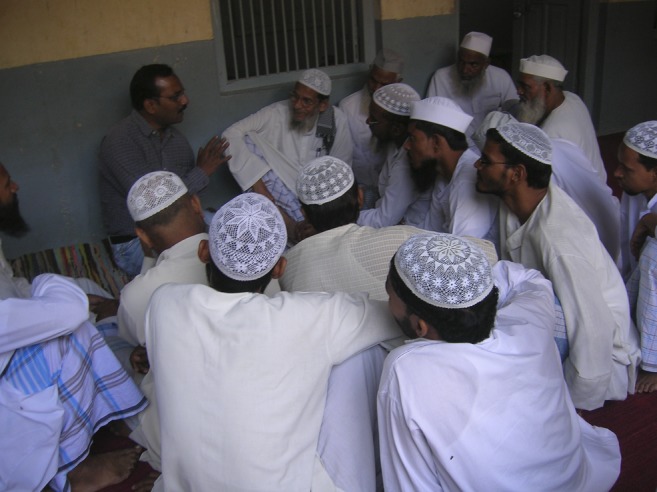
A religious discourse being held. Photo Credit: Thomson Thomas, India. This figure appears in color at www.ajtmh.org.

Community members asked whether the vaccine contained any material that was forbidden in their religious teachings or whether they were contradicting any religious dogma by repeatedly exposing their small children to the vaccine. The CGPP mobilizers held meetings with smaller groups of both male and female religious teachers, where positive references and supportive religious texts were highlighted. Connections to religious leaders made it easier to approach keepers of mosques and request announcements advising families to take their children to the booths. Friday sermons also began to carry the message that the polio vaccine was safe and protected children from becoming crippled (Figure 1).

**Figure 2.**
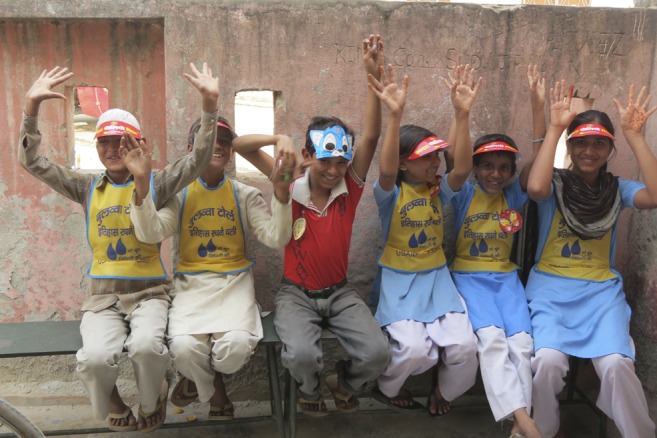
Children  forming *Bulawwa tolies* to promote participation in immunization campaigns. Photo Credit: Rina Dey, Behavior Change Communication Advisor. This figure appears in color at www.ajtmh.org.

Children were used as mobilizers, forming into groups called *Bulawwa tolies*.‖ These children were seen as nonthreatening and became enthusiastic ambassadors of change—carrying positive vaccination messages through communities, even bringing babies to booths for vaccination (Figure 2). Rotary International club members provided whistles, balls, flags, and masks as small rewards to child mobilizers, and their impact was far-reaching. They helped track small children, motivated their mothers, and, in many cases, even brought children to booths for the polio drops. Schoolchildren were used to hold rallies and parades a day before the round. Along with the schoolchildren, teachers also played a key role in mobilization. Most booths were set up in schools, where teachers were put on duty as vaccinators. Many gave up their Sundays to work at booths and even supervised the preparation of a mid-day meal to feed children who came to the booth.

Meetings with mothers and grandmothers proved easier than those with fathers, who were often working away from home during the hours of the immunization campaign or harbored strong resistance to the campaign. Some women would allow their children to be vaccinated but not let their doors to be marked for fear of their husband’s reaction. The mobilizers were tenacious and repeatedly visited the families to talk about polio and other childhood diseases and vaccinations. Slowly, community mobilizers and influencers formed bonds with resistant families, some of whom were migrant families who had never been reached by vaccination campaigns. It took many meetings and much persistence before families shared their fears, priorities, and problems. Mobilizers also formed links between families and frontline government workers such as Accredited Social Health Activists (ASHAs)¶ and ANMs. A major challenge was that the government community frontline workers often did not have the time or the inclination to provide answers to difficult queries. Frontline workers did not have the time or skills to explain to parents that repeated polio vaccination would not harm children, or that babies with 20 doses of polio vaccine could still get polio. Nor did they have the time or skills to allay their fears and answer their questions. The mobilizers filled this gap in many places and were able to provide the support and answers parents sought.

Communities often expressed concern over the lack of other health services in their communities. They wanted to know why the government was campaigning for only polio while their children were dying from other diseases. This gave rise to the idea of holding health camps in areas with very high number of resistors, just before the polio campaign rounds. Government medical officers were requested to send staff, drugs, and vaccines for the camp, and the CMCs organized the venue, provided transport to bring medical personnel, and sometimes even arranged for a female doctor to be contracted for a day so that antenatal and gynecological care could be provided. This became a very popular intervention, as it grew out of the stated need of communities and helped establish trust and goodwill among the CMCs, the CGPP, and the communities.

**Figure 3.**
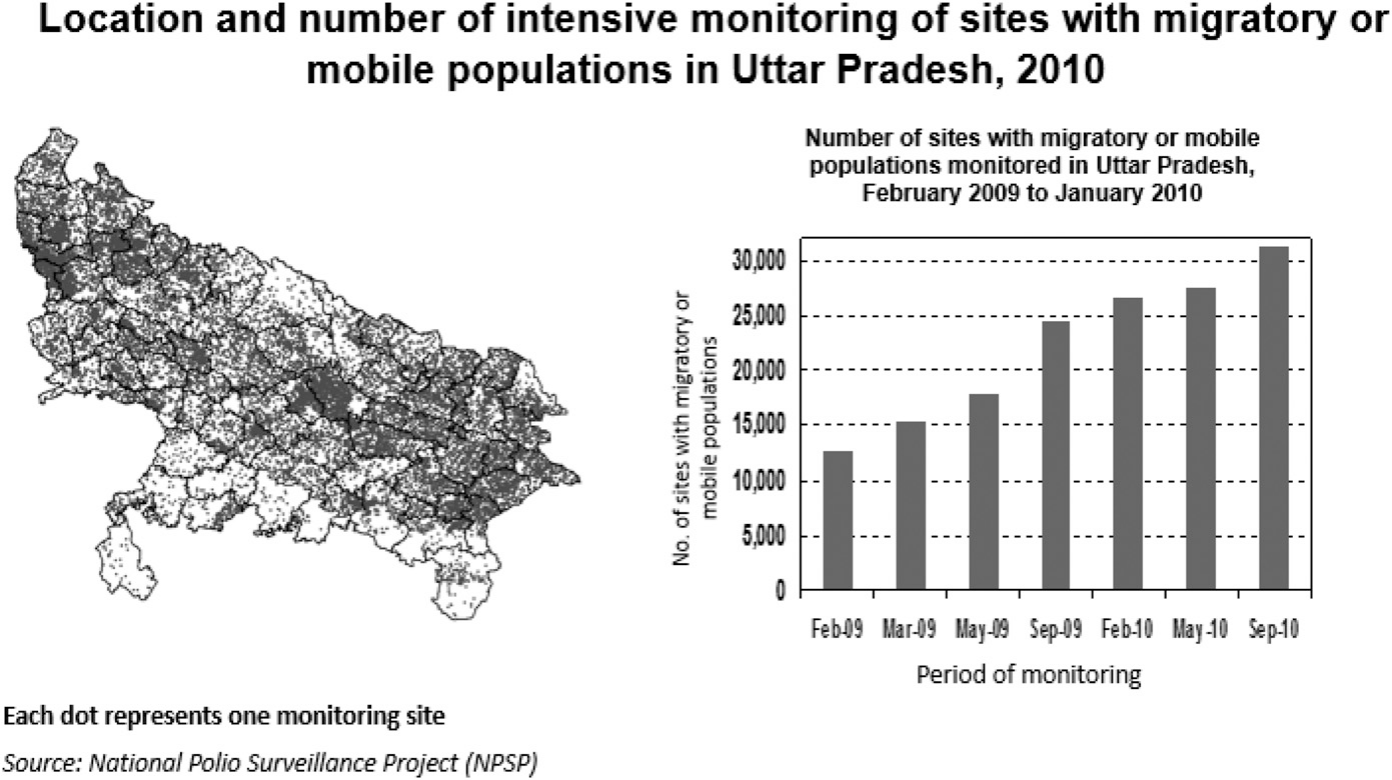
Location  and number of intensive monitoring of sites with migratory or mobile populations in Uttar Pradesh, 2010.^7^

India has a very large migrant population, and these families travel throughout the country looking for work. The CGPP created detailed maps to identify migrant sites and high-risk areas so that no family was in danger of being missed (Figure 3). Interstate liaising was created between government officials to track eligible children who were not registered anywhere.

Nomadic groups provided a special challenge because one or more families often appeared in high-risk areas overnight, making them increasingly difficult to track. Their children were often never registered in any health post and could have conceivably missed all vaccinations, including even polio rounds. Ration shop# owners, barbers, or others in the community who were likely the first to notice the appearance of new migrant families cooperated as informers, who were unpaid volunteers. They were trained to notify mobilizers, who in turn made sure that the children received immunization cards and vaccinations. Language barriers were common among nomads, so simple pictorial messages were used to convince these groups to vaccinate their children. 

Work with the community led to the identification of another gap in vaccination services. The state of Uttar Pradesh is home to a large brick kiln industry, drawing entire villages to migrate there for seasonal work. Children of brick kiln workers were largely bereft of all health care, and parents did not get leave from work for health-care visits or vaccination days. Workshops were held with the brick kiln owners who were sensitized and who provided lists of eligible children to the community mobilizers, who, in turn, ensured that all children were vaccinated during polio rounds.

In addition, famous Bollywood stars were recruited by the government and contributed to a very concerted and focused mass media program. Actors, cricketers, and other well-known leaders all joined in a chorus to stress the importance of “*Do boond zindagi ki*” (two drops of life). India’s leading superstar, Mr. Amitabh Bachchan, can take sole credit for converting many resistant families just by crooking his finger and asking why they had not given their children the “Two drops of life.”

These continuous multiple, targeted, multifaceted, and simultaneous approaches paid dividends. Most community members eventually relented and actively participated in the polio campaigns. However, instances of resistance continued to emerge, and some villagers used the program to negotiate with government functionaries, demanding better roads, electricity, and so forth. In some places, systemic failures of public services had to be addressed before the polio round could continue. For example, the district administration had to give in to demands for ration cards that entitle people to avail of the government’s public distribution system of food, for cleanliness drives, and so forth.

## LESSONS LEARNED

More than two decades of personal experiences have led to the following deductions that can have bearings on future public health programs. Success did not come easy for the CGPP. However, strong sustained efforts along with both successful and failed strategies led to many valuable lessons, the first and foremost being that civil society must never be taken for granted. Even the least empowered, poorest, and most illiterate people are blessed with wisdom and, therefore, have the right to facts.

The next step in the national polio eradication program took people by surprise when vaccinators started appearing on their doorsteps with increasing frequency. In underserved areas where routine immunization and other health services were wholly inadequate, this gave rise to suspicion about the vaccine and about the government’s intentions. To be fair, the resistance came as a big surprise even to the frontline workers who were not trained to handle this kind of behavior. It also strained their capacity to deliver routine services.

Program implementers must have their fingers on the pulse of the people and should operate with transparency. All queries and concerns must be addressed honestly and without delay. Failing to address pertinent issues directly can lead to misinformation and distrust that can snowball into a crisis. As bad news travels faster than good news, all adverse reports in the media need to be countered immediately before they spread deeper into the community.

One example of queries and concerns that were not addressed early on is the following: Did anyone bother to explain to parents that repeated polio vaccine doses would not harm their children? Or, why babies with 20 doses would still get polio? In India, it is to the credit of community mobilizers and the communities that, in time, parents did start to cooperate and accept the polio vaccine even when vaccinators appeared repeatedly on their doorsteps and/or intercepted parents while they were travelling with their children.

In states where the government health services were good, there was not much of a problem, and poliovirus transmission stopped quite early. But in states such as Uttar Pradesh, especially in its western districts with poor indicators, the wild poliovirus managed to take advantage of the weakness of the health system and infect children who were unimmunized or had weak immunity.

All members of civil society need to be treated with respect and must be heard. This is easier said than done because listening is an art and needs to be cultivated and must lead to hearing. Interpersonal communication training, particularly for frontline workers, was invaluable and allowed them to hear and react to the needs of their communities. Observing their body language, not engaging in counterproductive arguments, and treating families (especially resistant families) with respect—all these skills proved the key to success and took time to cultivate.

This attitudinal change among frontline health workers must be maintained and become a part of their curriculum. Monitoring and supportive supervision go hand-in-hand with success. Many public health programs fail because of weak supervision or failing to provide hands-on support where needed. The polio eradication program also began the same way. Workers were punished and suspended for reporting possible polio cases, or for not being able to achieve good immunization coverage at their booths or even during house-to-house visits. But when punishment turned to support and mentoring, the supervisors and those who were supervised became able to devise constructive strategies.

In hindsight, communication was one of the keys to success. It is likely that if communication strategies had been better planned well in advance, there would have been less skepticism from the people and acceptance of polio immunization would have been less problematic.

For many at-risk families, survival is the foremost priority, and government health services are not very accessible. The polio vaccination program should have been introduced in an environment of mutual understanding to gain community involvement instead of through a vertical, top-down approach. This does not imply that it was totally devoid of planning. The technical components—vaccine availability, cold chain strengthening, microplanning, surveillance for acute flaccid paralysis, and laboratory upgrades—were devised with deliberate precision and careful thought. What the planners underestimated, and therefore missed, was the lack of planning to ensure that the concerns of the population are addressed before and during the campaign. Responding to their needs for information, questions, and concerns, particularly when related to the health and future of their children, should have been the first step.

Another issue is that governments sometimes equate civil society with activist NGOs that are not accountable to anybody. Any mutual distrust had to be put aside for the program to be successful, and in the end, a partnership and healthy respect were developed, taking into account each other’s abilities and limitations. A program of this complexity must be inclusive and not coercive. The new and stronger relationship and trust between civil society and the government need to be nurtured and not allowed to fade away.

There is hardly any program in the world that has generated so much data as the Global Polio Eradication Initiative. The stakeholders learnt to base their strategies on data and modify them. Something that worked in one community might not work in another, and what worked today may not work tomorrow. This agility to change strategies midstream proved a key strength of frontline workers, including community mobilizers, who were constantly tasked with addressing emerging barriers and issues. Messaging required constant adjustment to respond to rumors, misinformation, and national and world events that impacted the program. Truth and constant sharing of correct information were the keys to combatting anti-vaccine literature and other forms of misinformation. Sharing of appropriate data with community members helped to allay and disprove these claims.

Very early on in the program, we all learned that there was no “one size fits all” solution. Families living next door to each other sometimes had to be spoken to differently, and their participation in the program could never be taken for granted. Each *madrassa*†† and each religious leader or priest had to be approached differently. The SMNet hired people to do just this task. These people were called district underserved coordinators. The district underserved coordinators focused on building an effective liaison with leaders of underserved communities. The coordinators were given special training on the religious practices of these communities, and they developed a good rapport with institutions in these communities such as *madrassas*. They equipped themselves with statements from religious texts regarding the health and well-being of children so that they could dialogue with the very learned faculty members of the various religious schools of thought in these communities.

Local media too must be treated with great respect and care because they are more important than national media for the program. Using them to spread correct information is invaluable. In this case too, the local press helped to create ownership for community members. The CGPP community mobilizers sensitized representatives of the local media and were able to act as sources of truth. With support from polio partners such as UNICEF, Rotary International, and the WHO, media sensitization workshops were organized from time to time in selected districts and at the state level.^8^

The CGPP found that there was a lack of awareness about the importance of routine childhood immunization. If mothers had known about its importance and regularly followed the immunization schedule, there would have been higher levels of immunity among their children and less resistance to polio immunization. An increased emphasis on routine immunization must follow as a natural corollary, and this is the time to strike when doors are open and when people have started to believe in government health services. For parents to take immunization as a natural part of bringing up their children, immunization programs must be of the highest quality and regular. Just as giving polio drops to their children every time there was a polio round, the receipt of other vaccines as the schedule calls for must also become a habit.

Last, but not least, the most valuable lesson that all program partners learnt was to work together seamlessly. It did not start out that way, but in the face of so much resistance, there was no option but to band and bond together and to stop blaming each other. Solutions were thought of jointly. Each partner knew its own role and played its part as best it could. Acknowledging the government’s captaincy, its request was taken as a command and no partner ever refused to tackle an issue, no matter how difficult it was.

This strategy should pave the way for future programs. Efforts must be complementary, not competitive. Roles need to be defined based on partner competencies: for example, technical agencies are best equipped to provide technical guidance, whereas community-based organizations are skilled in communication and behavior change.

As all sectors of society contributed their best efforts to make the program a success, the program finally turned into a people’s movement. Success came finally when polio transmission ended, and India was certified polio-free in March 2014—a hard-won fight but rich in lessons for the future. In 2012, at the request of the district government administration, a large installation commemorating civil society’s contribution was placed in the middle of a busy thoroughfare in Moradabad, Uttar Pradesh, a town once considered the epicenter of polio transmission in the world (Supplemental Figure 1a). The administration also requested the establishment of a polio museum in the district hospital. A polio gallery was made that tracked the history of how polio was eradicated from India. In July 2014, a village headman, using his own unrestricted funds, erected a huge gate called the Polio Eradication Gate in Jansath, Muzaffarnagar, a district in Uttar Pradesh that had had many polio cases in the not-too-distant past (Supplemental Figure 1b).

India’s polio eradication program is a shining example of what can be achieved if civil society is brought into the inner circle of planning and policy making and if each partner’s contribution is recognized and respected. The work must not stop here but needs to be continued with the same, if not even stronger, momentum and commitment toward the remaining public health challenges of our time.

## Supplemental figure

Supplemental materials

## Footnotes

† † A *fatwa* is a ruling on a point of Islamic law given by a recognized authority.

‡ ‡ Forbidden or proscribed by Islamic law.

§ § The chief Imam of one of India’s largest mosques.

‖ ‖ Small groups of children identified and managed by CMCs, who went from house to house to call mothers to bring their babies to polio booths.

¶ ¶ Accredited social health activists, a government community health worker cadre.

# # Shops selling subsidized food grains, oils, and other basic good necessities through the public distribution system.

†† †† A school where children are also taught about Islam.

## References

[1] GPEI, 2012 A Year without Polio in India. Available at: http://polioeradication.org/news-post/a-year-without-polio-in-india/. Accessed April 18, 2019.

[2] GPEI, 2017 India Celebrates Six Years Polio Free. Available at: http://polioeradication.org/news-post/india-celebrates-six-years-polio-free/. Accessed April 18, 2019.

[3] Singh P Das J Dutta P, 2010 Misconceptions/rumors about oral polio vaccine in western UP: a case study. Indian J Prev Soc Med 41: 28–32.

[4] Losey L 2019 The CORE Group Polio Project: an overview of its history and its contributions to the global polio eradication initiative. Am J Trop Med Hyg 101 (Suppl 4): 4–14. 10.4269/ajtmh.18-0916 PMC6776098 31760971

[5] Choudhary M Perry H Solomon R, 2019 Effectiveness of a census-based management information system for guiding polio eradication and routine immunization activities: evidence from the CORE Group Polio Project in Uttar Pradesh, India. Am J Trop Med Hyg 101 (Suppl 4): 33–44. 31760973 10.4269/ajtmh.18-0935 PMC6776090

[6] Awale J Choudhary M Solomon R Chaturvedi A, 2019 Effective partnership mechanisms: a legacy of the polio eradication initiative in India and their potential for addressing other public health priorities. Am J Trop Med Hyg 101 (Suppl 4): 21–32. 10.4269/ajtmh.18-0938 PMC6776101 31760982

[7] Bhagwat P, 2010 Progress and Challenges in Polio Eradication: Uttar Pradesh. 22nd India Expert Advisory Group (IEAG) Meeting, Delhi, India, November 1, 2010.

[8] Galway M, 2005 Communication Update. India Expert Advisory Group Meeting Global Polio Eradication Initiative, New Delhi, India, May 3, 2005.

