# Supplementary material for: Involvement of Civil Society in India’s Polio Eradication Program: Lessons Learned
Source: Am J Trop Med Hyg. 2019 Oct;101(4 Suppl):15–20. doi: 10.4269/ajtmh.18-0931 (PMC6776100; doi:10.4269/ajtmh.18-0931)
Supplement: Supplementary file 1 [file tpmd180931.SD1.pdf]

The following are supplemental materials and will be published online only

# 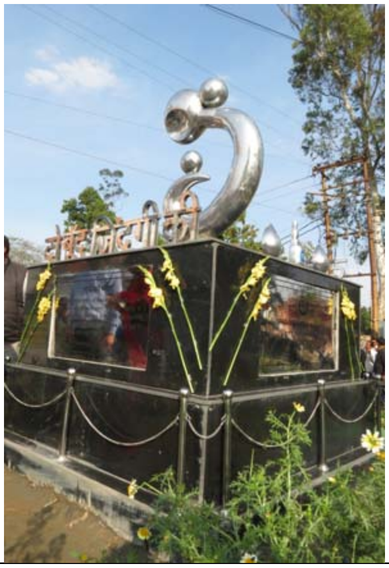	# 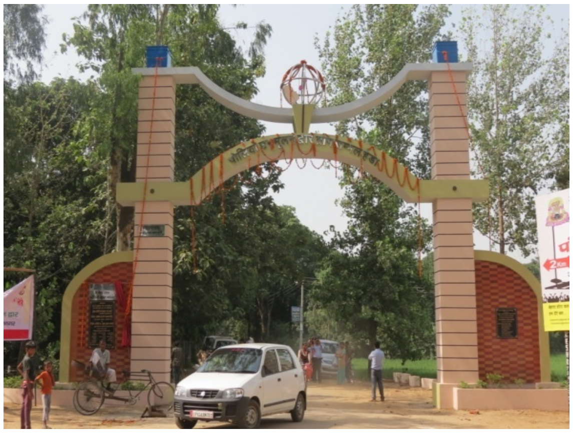
**Polio chowk*, Moradabad (Fig. 1a)**	**Gate commemorating the eradication of polio, Muzaffarnagar (Fig. 1b)**
**Supplemental Figure 1. Memorials celebrating the eradication of polio**	

*A chowk is a prominent road crossing.

#
